# Immunoproteasome inhibition attenuates experimental psoriasis

**DOI:** 10.3389/fimmu.2022.1075615

**Published:** 2022-12-14

**Authors:** Marta del Rio Oliva, Mark Mellett, Michael Basler

**Affiliations:** ^1^ Division of Immunology, Department of Biology, University of Konstanz, Konstanz, Germany; ^2^ Department of Dermatology, University Hospital Zürich (USZ), Zürich, Switzerland; ^3^ Faculty of Medicine, University of Zürich (UZH), Zürich, Switzerland; ^4^ Biotechnology Institute Thurgau at the University of Konstanz, Kreuzlingen, Switzerland

**Keywords:** immunoproteasome inhibition, psoriasis, CARD14, imiquimod, ONX 0914

## Abstract

**Introduction:**

Psoriasis is an autoimmune skin disease associated with multiple comorbidities. The immunoproteasome is a special form of the proteasome expressed in cells of hematopoietic origin.

**Methods:**

The therapeutic use of ONX 0914, a selective inhibitor of the immunoproteasome, was investigated in *Card14ΔE138^+/-^* mice, which spontaneously develop psoriasis-like symptoms, and in the imiquimod murine model.

**Results:**

In both models, treatment with ONX 0914 significantly reduced skin thickness, inflammation scores, and pathological lesions in the analyzed skin tissue. Furthermore, immunoproteasome inhibition normalized the expression of several pro-inflammatory genes in the ear and significantly reduced the inflammatory infiltrate, accompanied by a significant alteration in the αβ^+^ and γδ^+^ T cell subsets.

**Discussion:**

ONX 0914 ameliorated psoriasis-like symptoms in two different murine psoriasis models, which supports the use of immunoproteasome inhibitors as a therapeutic treatment in psoriasis.

## 1 Introduction

Psoriasis is a chronic autoimmune disorder that affects 2-3% of the general population (1). It is characterized by increased keratinocyte proliferation (2), resulting in the formation of red and scaly plaques. Topical treatments, including corticosteroids, are often discontinued due to their numerous side effects (3). It is currently accepted that the disorder is mediated by the cross-talk between epidermal keratinocytes and immune cells (4). Indeed, psoriatic keratinocytes can activate neutrophils, plasmacytoid dendritic cells, and T cells (5), that aberrantly proliferate in response to inflammatory cytokines such as interleukin-22 (IL-22), and IL-17A (6).

The complexity of this disease has hampered the development of new therapies due to difficulties mimicking human psoriasis in animal models (7). Next-generation sequencing of patients with familial psoriasis revealed a gain-of-function mutation in the caspase recruitment domain family member 14 (*CARD14*) (8). Heterozygous mice harboring a CARD14 gain-of-function mutation (*Card14ΔE138* ^+/-^) spontaneously develop a chronic psoriatic phenotype with scaling skin lesions (9). Several other murine models induce psoriasis-like features (10). Topical application of imiquimod (IMQ), a TLR7/8 activator, induces skin inflammation mediated *via* the IL-23/IL-17A axis (11). Psoriatic lesions depict an upregulation in retinoic acid-related orphan receptor C (RORC) mRNA (12), which controls the lineage commitment of T helper type 17 (Th17) (13). The increase of IL-17A and IL-22 in serum samples of psoriasis patients (14) demonstrates that its pathogenesis is driven by the IL-23/IL-17A axis. Furthermore, the neutralization of cytokines that maintain Th17 cell polarization reduces skin lesions (9).

The immunoproteasome is a special form of the 26S proteasome in which the standard catalytically active β-subunits (β1c, β2c, and β5c) are replaced by low molecular mass polypeptide (LMP)2 (β1i), multicatalytic endopeptidase complex-like (MECL)-1 (β2i) and LMP7 (β5i). The expression of both standard proteasome and immunoproteasome subunits is increased in lesional psoriasis skin (15). The immunoproteasome is not only involved in the generation of antigenic peptides that are presented to cytotoxic T cells (16) but has a strong influence on T helper cell commitment (17). Immunoproteasome inhibition is a promising strategy in reducing IL-23 secretion and suppressing Th17 cell development (18). Irreversible inhibition of the LMP2/LMP7 subunits of the immunoproteasome *via* treatment with ONX 0914 has been demonstrated to ameliorate several inflammatory diseases (19–22).

In this study, the therapeutic potential of immunoproteasome inhibition in psoriasis pathogenesis was assessed in Card14-mediated and IMQ-induced psoriasiform models. We found disease amelioration in two different pre-clinical psoriasis models, which suggests selective inhibition of the immunoproteasome as a potential therapeutic treatment strategy for psoriasis.

## 2 Materials and methods

### 2.1 Mice

C57BL/6 mice (H-2^b^) were originally obtained from Charles River Laboratories. *Card14ΔE138* ^+/-^mice were originally described in (9). Naïve C57BL/6 mice were used as negative controls for *Card14ΔE138* ^+/-^mice. IL-17A-GFP (C57BL/6-Il17atm1Bcgen/J; stock #018472 (23)) mice were purchased from The Jackson Laboratories. The animal study was reviewed and approved by Regierungspräsidium Freiburg (G-20/20).

### 2.2 Murine models and proteasome inhibition

ONX 0914 (Kezar Life Sciences) was formulated in 10% sulfobutylether-β-cyclodextrin and 10 mM sodium citrate (pH 6; vehicle) (19). The administration was performed s.c. at 10 mg/kg, which has extensively been used in the past not causing cytotoxic effects even at a higher concentration (12 mg/kg) (21). The activity of the proteasome after the use of ONX 0914 was previously investigated (19, 24). In the IMQ-induced psoriasis-like model, IL-17A-GFP mice were shaved on the back and 5% IMQ cream (Aldara, MEDA) was applied to the back and the ear daily for 8 consecutive days. Starting on day 3, mice were treated daily with ONX 0914 or vehicle s.c. Experiments with *Card14ΔE138* ^+/-^ mice started at the age of 8-10 weeks. Mice were treated with ONX 0914 or vehicle s.c. on alternate days for 20 days.

### 2.3 Ear thickness and skin inflammation score

Ear thickness was measured (thickness gauge; Mitutoyo) daily or on alternate days in the IL-17A-GFP and *Card14ΔE138* ^+/-^ mice, respectively. Eczema and scaling on the ear and back were evaluated visually in a blinded manner and quantified on a range from 0 to 4 points (0, no change; 1 mild change, 2 marked change, 3 significant change, 4 severe change). The inflammation score represents the sum of both factors.

### 2.4 Real-time RT-PCR

RNA was extracted from the ear tissue using Trizol (ThermoFisher) according to manufacturer´s protocols. The cDNA was prepared using the Biozym cDNA conversion kit (Biozym). Afterwards, real-time RT-PCR (Biozym Blue S´Green Kit) was performed in a Biometra TProfessional Thermocycler (Analytik Jena). The primers used are listed in **Supplementary Table 1**.

### 2.5 Histology

Hematoxylin-eosin sections were prepared as in (22). For immunofluorescence staining, the samples were flash-frozen in liquid nitrogen and embedded into anOptimal cutting temperature compound (OCT) medium. Sections of 14 μm were prepared using the Frigocut 2800E (Reichert Jung/Leica) and were hydrated in PBS at RT for 10 min. The samples were fixed with acetone at 4°C for 15 min and washed in PBS. Staining (listed in **Supplementary Table 2**) was performed overnight at 4°C. Counterstaining was performed with DAPI mounting medium (ThermoFisher). Images were taken in AxioImager (Zeiss). Quantification of the epidermal thickness was performed in ImageJ (U.S. National Institutes of Health) as described in (25). Quantification of the immune populations infiltrating the ear was performed by measuring the percentage of the positive area and normalizing it to DAPI with ImageJ.

### 2.6 Organ preparation and flow cytometry

Spleens were collected and a single cell suspension was prepared using 70 µm nylon mesh. Ears were harvested and dorsal and ventral sections were split with forceps. Digestion was performed with 1 mg/ml DNAse I (Sigma) and 1 mg/ml collagenase D (Roche) in HBSS (10 mM Hepes) in a gentleMACS Octo Dissociator (Miltenyi Biotec). Cytokine production was analyzed after restimulation with 25 ng/ml phorbol-12-myristat-13-acetat (PMA), 500 ng/ml ionomycin and 10 µg/ml brefeldin A (BFA) (all Merck) for 4 hours at 37°C, 5% CO2. Surface and intracellular staining was performed as in (26). Doublet exclusion was performed by gating on SSC-W/SSC-H or SSC-H/SSC-A. The surface staining was performed first along with fixable viability stain 780 (BD Pharmingen) according to the manufacturer’s instructions. The antibodies used are listed in **Supplementary Table 2**. The samples were measured on LSRFortessa (BD Biosciences). Cell count in the ear was performed using Cytoflex (Beckman coulter). Flow cytometry data was analyzed with FlowJo v10 (BD Biosciences).

### 2.7 Serum collection and enzyme-linked immunosorbent assay (ELISA)

Blood was collected by cardiac puncture. The analysis of IL-17A, IL-6 and TNF (ThermoFisher Scientific) was performed as in (22).

### 2.8 Statistics

Data is expressed as mean ± S.D and was analyzed using Prism 9.1 (Graphpad). The Shapiro-Wilk (W) test was used to verify normal distribution. Data without a normal distribution were analyzed with non-parametric tests (Kruskal-Wallis or Mann-Whitney test), and data with a normal distribution were analyzed with parametric tests (unpaired t-test, Ordinary one-way or two-way ANOVA), including the *post hoc* test Bonferroni, Tukey, Šidák or Fisher´s LSD. Statistical significance was achieved when p < 0.05; * p < 0.05, ** p < 0.01, *** p < 0.001, and **** p < 0.0001.

## 3 Results

### 3.1 Immunoproteasome inhibition attenuated psoriasis-like lesions in Card14ΔE138^+/-^ mice

**Figure 1:**
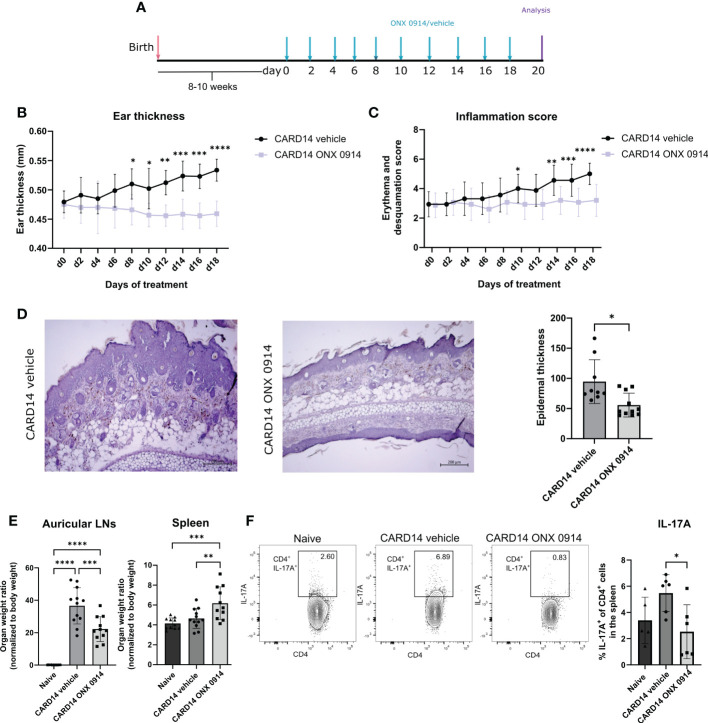
Immunoproteasome inhibition attenuated the psoriasis-like lesions in *Card14ΔE138* ^+/-^ mice: 8-10 weeks old *Card14ΔE138* ^+/-^ mice were treated on alternate days with 10 mg/kg ONX 0914 or vehicle for 20 days. **(A)** Experimental setup. **(B)** Ear thickness was measured with a thickness gauge. On the γ-axis, the ear thickness in mm is depicted. Data (vehicle n = 7, ONX 0914 n = 6) was pooled from two independent experiments and analyzed by a two-way ANOVA followed by a Šidák test. **(C)** The inflammation score was measured visually on alternate days and results from the sum of the eczema and scaling scores, which are shown on the γ-axis. Data (vehicle n = 16, ONX 0914 n = 15) was pooled from five independent experiments and analyzed by a two-way ANOVA followed by a Šidák test. **(D)** Representative hematoxylin-eosin-stained sections from the ear of *Card14ΔE138* ^+/-^ after 20 days of treatment with ONX 0914 or vehicle. The epidermal thickness was measured in ImageJ and normalized to the epidermal area, which is depicted on the γ-axis. Data (vehicle n = 9, ONX 0914 n = 11) was pooled from three independent experiments and analyzed by a Mann-Whitney test. The scale bar is 200 μm. **(E)** The auricular lymph nodes and the spleens were harvested after 20 days of ONX 0914 treatment and weighed. On the γ-axis, the organ-to-body weight ratio is depicted. Naïve mice were used as controls. Data (naïve n = 11, vehicle n = 12, ONX 0914 n = 11) was pooled from three independent experiments and analyzed by one-way ANOVA followed by a Tukey´s test. **(F)**. The splenocytes of mice treated with ONX 0914 or vehicle were collected and stimulated with PMA, ionomycin and BFA for 4 hours at 37°C. Then, an intracellular cytokine staining for IL-17A was performed. On the γ-axis the frequency of IL-17A^+^ cells in the spleen is depicted (left panel). The gating strategy is depicted in **Supplementary Figure 3A** and includes doublet and dead cell exclusion. The IL-17A^+^ cells are pregated on CD45^+^ CD4^+^ cells. Gating was performed using a Fluorescence minus one (FMO) control. Representative dot plots are depicted on the right panels. Data (n = 6) was pooled from two separate experiments and analyzed by one-way ANOVA followed by a Tukey´s test. All values represent mean ± SD. * p < 0.05, ** p <0.01, *** p < 0.001, and **** p < 0.0001.

*Card14ΔE138* ^+/-^ mice develop spontaneous ear skin lesions at approximately 8 weeks of age that mimic human psoriasis. To investigate the potential therapeutic use of ONX 0914 we treated *Card14ΔE138* ^+/-^ mice at the age of 8-10 weeks with 10 mg/kg ONX 0914 on alternate days for 20 days (**Figure 1A**). Mice treated with the immunoproteasome inhibitor depicted significantly decreased ear thickness and epidermal thickness compared to vehicle-treated mice (**Figures 1B, D**). Furthermore, the inflammation score was significantly decreased after treatment with ONX 0914 (**Figure 1C**). The hematoxylin-eosin sections of the ear demonstrated the presence of thickening epidermis (acanthosis) and thickened stratum corneum (hyperkeratosis) in *Card14ΔE138* ^+/-^ mice (**Figure 1D**). In contrast, treatment with ONX 0914 notably alleviated the histopathology features typical of psoriasis. We also observed an increase in the size of the draining lymph nodes (dLNs) collected from *Card14ΔE138* ^+/-^ vehicle-treated mice in comparison to naïve mice (**Figure 1E**). Even though the organ weight ratio of the dLNs after immunoproteasome inhibition was not reduced to basal levels of naïve mice, a significant reduction compared to vehicle-treated mice was detected. In contrast, ONX 0914-treated mice depicted a significantly increased weight of the spleen. The percentage of IL-17A^+^ cells were significantly reduced after treatment with ONX 0914 in both auricular and inguinal lymph nodes (**Supplementary Figure 1**) while the percentage of IL-22^+^ cells was not affected. We also investigated the presence of IL-17A-secreting CD4^+^ cells in the spleen (**Figure 1F**), which was significantly reduced in ONX-0914-treated mice. In contrast to IL-17A, the serum levels of TNF and IL-6 in *Card14ΔE138^+/-^* mice were elevated compared to naïve control mice. However, no difference in serum levels of TNF and IL-6 was observed between ONX 0914-treated and vehicle-treated mice (**Supplementary Figure 2**).

### 3.2 Expression patterns of psoriasis-related genes

**Figure 2:**
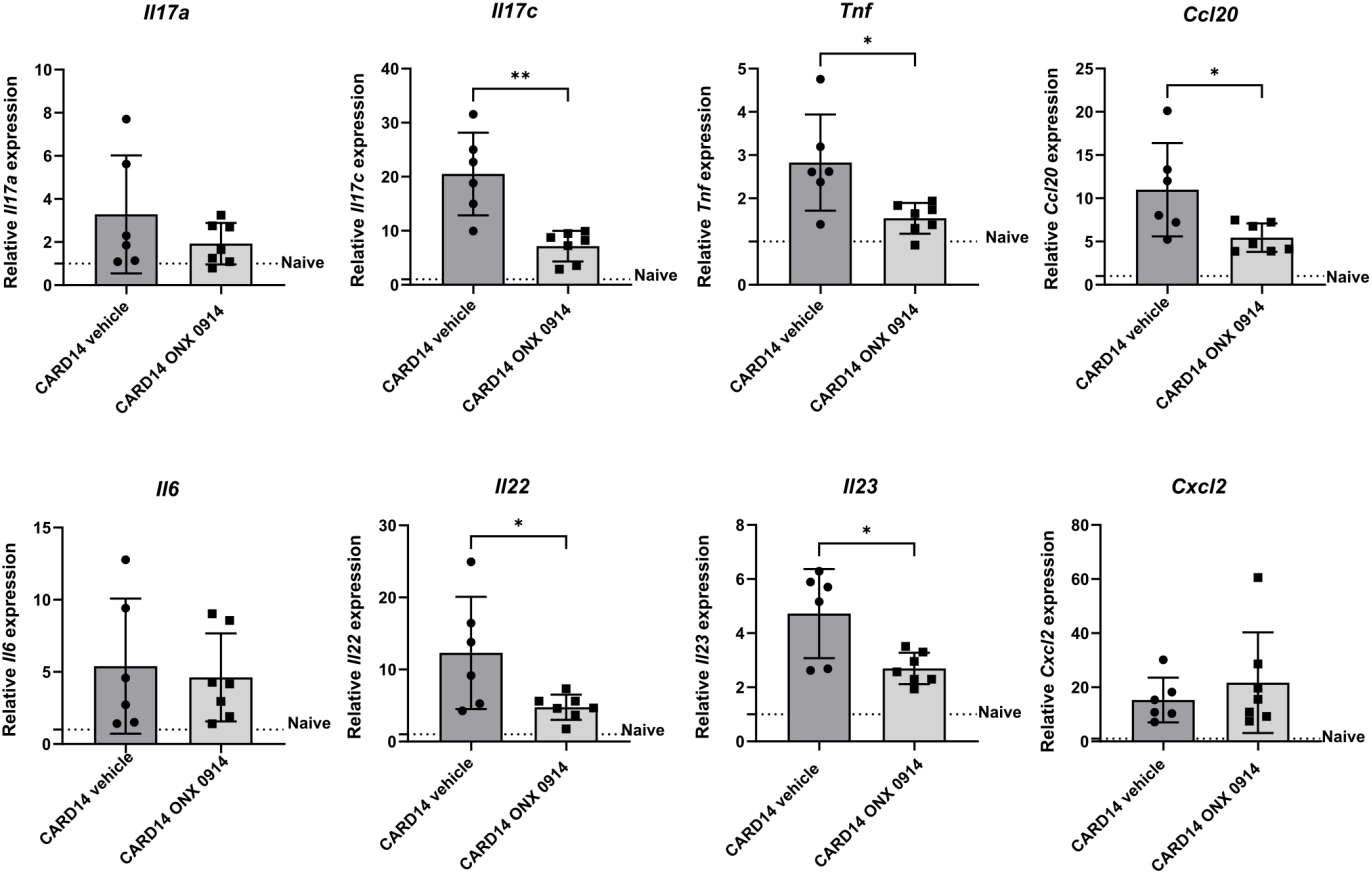
Immunoproteasome inhibition reduces the expression of inflammatory genes in *Card14ΔE138* ^+/-^ mice: 8-10 weeks old *Card14ΔE138* ^+/-^ mice were treated on alternate days with 10 mg/kg ONX 0914 (n = 7) or vehicle (n = 6) for 20 days. Real-time RT-PCR analysis of ear tissue was performed for *Il17a*, *Il17c*, *Tnf*, *Ccl20*, *Il6*, *Il22*, *Il23*, and *Cxcl2*. On the γ-axis, the relative expression of each gene is depicted. Naïve C57BL/6 mice were used as a control indicated by the dotted line. Data were analyzed following the 2^–ΔΔCt^ method and normalized to hprt. Data were pooled from 2 independent experiments and statistically analyzed by unpaired t-test. Values represent mean ± SD. * p < 0.05, and ** p <0.01.

To assess the changes in the inflammatory milieu, we determined the gene expression of several inflammatory mediators in the ear tissue of *Card14ΔE138* ^+/-^ mice (**Figure 2**). Compared to naïve wild-type mice, several inflammation-related genes were upregulated in *Card14ΔE138* ^+/-^ mice. Immunoproteasome inhibition significantly decreased the mRNA expression of the inflammatory mediators *Il17c*, *Tnf*, *Ccl20*, *Il22*, and *Il23*. No differences in the expression of *Il17a*, *Il6*, or *Cxcl2* were detected.

### 3.3 ONX 0914 reduces the inflammatory infiltration in the ear of psoriatic mice

**Figure 3:**
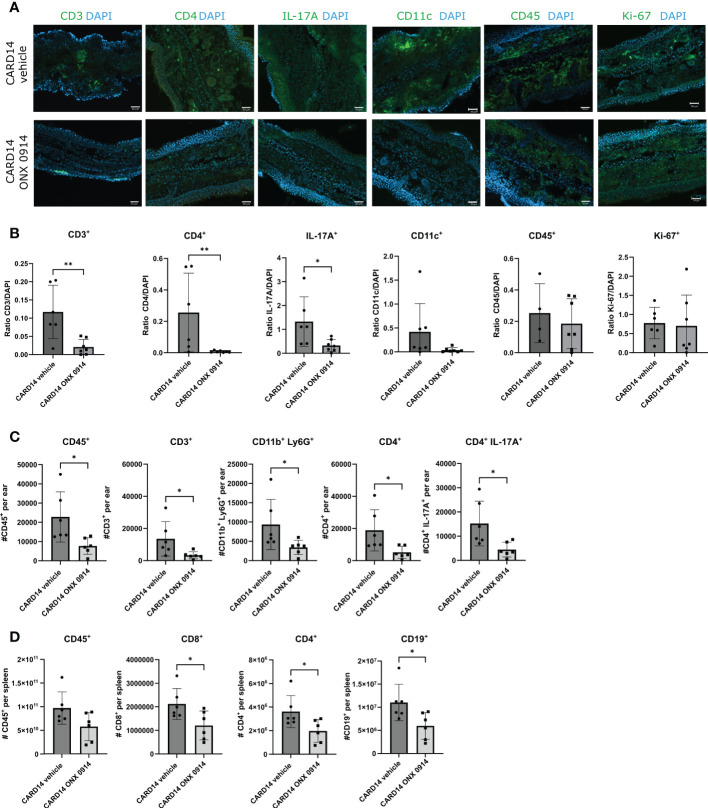
Immune cell populations in the ear and spleen of *Card14ΔE138* ^+/-^ mice: 8-10 week old *Card14ΔE138* ^+/-^ mice were treated on alternate days with 10 mg/kg ONX 0914 (n=6-7) or vehicle (n=4-7). 20 days after treatment, 14 μm ear cryosections were stained with anti-CD3, anti-CD4, anti-IL-17A, anti-CD11c, anti-CD45, anti-Ki67, antibodies (all in green) and DAPI (in blue). Representative images are shown. The scale bar is 50 μm **(A)**. The positive signals were quantified with ImageJ **(B)** On the γ-axis, the ratio of the fluorescence signal to DAPI is depicted. Data (n=4-7) were pooled from 2 independent experiments and statistically analyzed by unpaired t-test or Mann-Whitney test. **(C)** A single cell suspension of the ear (n=6) was prepared after 20 days of ONX 0914 or vehicle treatment and the CD45^+^, CD3^+^, CD11b^+^Ly6G^+^, CD4^+^, and CD4^+^IL-17A^+^ populations were analyzed. On the γ-axis, the absolute cell count per ear is depicted. **(D)** The spleen was analyzed for CD45^+^, CD8^+^, CD4^+^, and CD19^+^. The absolute cell number is depicted on the γ-axis. Data were pooled from 2 independent experiments (n=6) and statistically analyzed by unpaired t-test or Mann-Whitney test. **(C, D)** The cells were gated on CD45^+^ cells after doublet and dead cell exclusion. The gating strategy for C and D is depicted in **Supplementary Figure 3B** Representative flow cytometry plots for C and D are depicted in **Supplementary Figure 4A**. All values represent mean ± SD. * p < 0.05, and ** p <0.01.

Phenotyping the psoriasis inflammatory infiltrate revealed abundant mononuclear cells in the ear of *Card14ΔE138* ^+/-^ mice (**Figure 3**). We detected the presence of CD45^+^ and CD4^+^ cells distributed along the epidermis and dermis (**Figure 3A**). IL-17A seemed to be confined close to the epidermis. Quantification of the immunofluorescence signal in ear sections of *Card14ΔE138* ^+/-^ mice (**Figure 3B**) revealed that ONX 0914 treatment reduced the presence of CD3^+^ cells, CD4^+^ cells, and the pro-inflammatory cytokine IL-17A.

To confirm these results we investigated the inflammatory infiltrates in the ear by flow cytometry (**Figure 3C**). We observed a significant reduction in the absolute cell count of CD45^+^, CD3^+^, CD4^+^, CD11b^+^Ly6G^+^, and CD4^+^IL-17A^+^ in the ear of *Card14ΔE138* ^+/-^ mice treated with ONX 0914 and the reduction of CD8^+^, CD4^+^ and CD19^+^ in the spleen (**Figure 3D**). The reason for the apparent discrepancy between the observed increased spleen weight (**Figure 1E**) and the reduction of the numbers of CD8^+^, CD4^+^ and CD19^+^ in the spleen (**Figure 3D**) of ONX 0914 treated mice is currently unknown.

### 3.4 Immunoproteasome inhibition modulates the αβ^+^ and γδ^+^ T cell subsets

**Figure 4:**
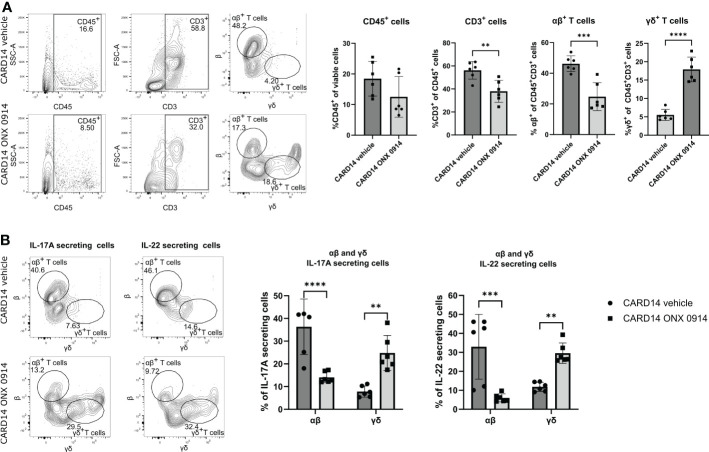
Immunoproteasome inhibition alters the αβ^+^ and γδ^+^ T cell subsets. 8-10 weeks old *Card14ΔE138* ^+/-^ mice were treated on alternate days with ONX 0914 (n = 6) or vehicle (n = 6) for 20 days. A single cell suspension of the ear was prepared and stimulated with PMA, ionomycin and BFA for 4 hours at 37°C. Then, an intracellular cytokine staining for IL-17A and IL-22 was performed. The αβ^+^ or γδ^+^ were gated on CD45^+^CD3^+^ cells. **(A)** Quantification of the frequencies of the analyzed cells. On the γ-axis, the percentage of the indicated population of viable or of CD45^+^CD3^+^ cells is depicted. Representative flow cytometry plots are shown on the left panel. αβ^+^ and γδ^+^ cells were gated on CD45^+^ CD3^+^ cells after doublet and dead cell exclusion. **(B)** IL-17A and IL-22 secreting αβ^+^ or γδ^+^ T cells. Representative flow cytometry plots are shown on the left panel. αβ^+^ and γδ^+^ cells were gated on CD45^+^ and IL-17A^+^ or IL-22^+^cells after doublet and dead cell exclusion. Data were pooled from two independent experiments and analyzed by unpaired t-test **(A)** or two-way ANOVA followed by a Šidák test **(B)**. The gating strategy is depicted in **Supplementary Figure 3D**. Gating was performed using FMO spleen samples. IL-17A and IL-22 secretion was gated using FMO ear samples. All values represent mean ± SD. * p < 0.05, ** p <0.01, *** p < 0.001, and **** p < 0.0001.

Skin homeostasis is maintained by balancing keratinocyte proliferation and destruction (27). In the past, most of the T cell functions have been attributed to αβ^+^ T cells, while γδ^+^ T cells have been overlooked (28). Therefore, we analyzed the presence of αβ^+^ and γδ^+^ T cells subsets in the ear. We observed that inhibition of the immunoproteasome in *Card14ΔE138* ^+/-^ mice induced a change in the T cell pool by decreasing the percentage of αβ^+^ T cells and increasing the γδ^+^ T cells (**Figures 4A,B**).

Both dermal αβ^+^ and γδ^+^ T cells can secrete IL-17A and IL-23 (29), which has been linked to the pathogenesis of psoriasis (30). To dissect the cellular source of IL-17A we analyzed the secretion of IL-17A and IL-22 cytokines by αβ^+^ and γδ^+^ T cells in the ear tissue of *Card14ΔE138* ^+/-^ mice after a short re-stimulation *in vitro*. While approximately 40% of the IL-17A-secreting cells in the ear were αβ^+^ T cells, ONX 0914 significantly decreased the secretion of IL-17A by these cells. IL-22 secretion was reduced as well in ONX 0914 treated mice (**Figure 4B**). This shift in IL-17A and IL-22 secretion suggests that immunoproteasome inhibition shapes the immunological response, causing an alteration in the cell subsets.

### 3.5 ONX 0914 ameliorates the skin lesions in the IMQ-induced psoriasis-like mouse model

**Figure 5:**
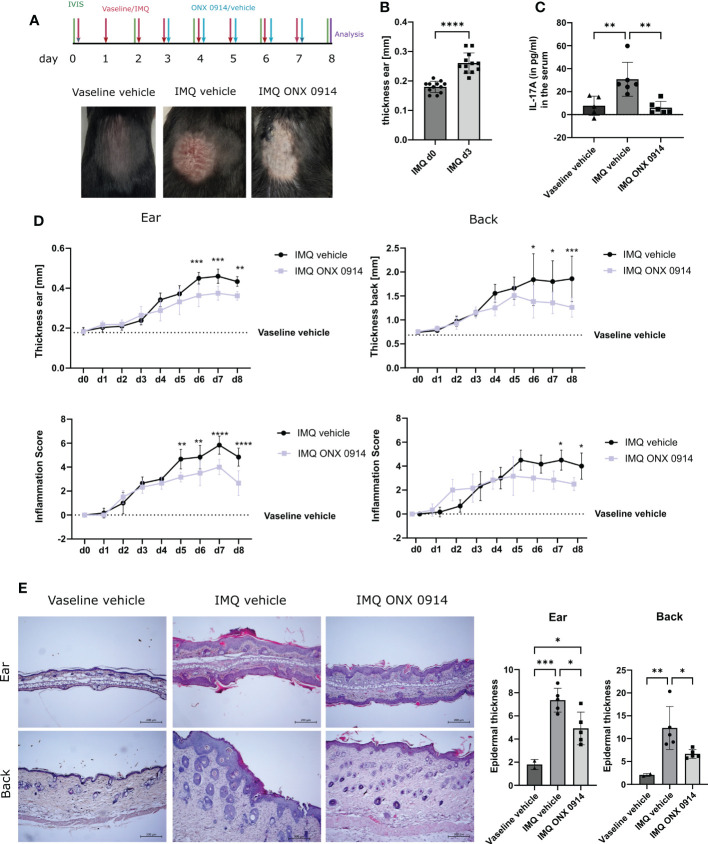
Immunoproteasome inhibition ameliorates IMQ-induced psoriasis-like inflammation in mice. IL-17A-GFP mice were treated with IMQ or vehicle (vaseline) on the back and the ear for 8 consecutive days. **(A)** Experimental setup and representative images of the back of the mice after 8 days of IMQ application. **(B)** Ear thickness in mm on day 0 and day 3 of IMQ-treated mice (n = 12). Data were pooled from three independent experiments and analyzed by paired t-test. **(C)** IL-17A levels in the serum of mice after 8 days of treatment. Data (vaseline vehicle-treated mice n = 5, IMQ vehicle and IMQ ONX 0914 n = 6) was pooled from two independent experiments and analyzed by one-way ANOVA followed by a Tukey´s test **(D)** Ear and back thickness were measured with a thickness gauge. On the γ-axis, the ear thickness in mm is depicted. The thickness from the vaseline vehicle group is depicted for clarification (dotted line) and was not statistically analyzed. The inflammation score was measured visually on alternate days and results from the sum of the eczema and scaling scores, which is shown on the γ-axis. Data (IMQ vehicle n = 6, IMQ ONX 0914 n = 6) was pooled from two independent experiments and analyzed by a two-way ANOVA followed by a Šidák test. **(E)** Representative images of hematoxylin-eosin stained sections from the ear and back of vaseline-vehicle, IMQ-vehicle, or IMQ-ONX 0914-treated mice. The scale bar is 200 μm. Epidermal thickness from the ear and the back was calculated using ImageJ. Data (vaseline vehicle n = 2, IMQ vehicle, and ONX 0914 n = 5) was pooled from two independent experiments and analyzed by one-way ANOVA followed by a Tukey´s test. All values represent mean ± SD. * p < 0.05, ** p <0.01, *** p < 0.001, and **** p < 0.0001.

To validate our findings in another murine model of psoriasis, we analyzed the effect of immunoproteasome inhibition in the IMQ-induced psoriasis-like mouse model, which is an acute psoriasiform model. To ensure proper immunoproteasome inhibition in the acute psoriasis-like mouse model ONX 0914 was administered daily instead of every second day as applied in the *Card14ΔE138^+/-^* mice. To easily track IL-17A-secreting cells, IL-17A-GFP reporter mice were used. IL-17A-GFP mice received daily IMQ or vaseline cream applied on the back and the ear for 8 consecutive days (**Figure 5A**). ONX 0914 or vehicle was administered starting on day 3, a time point when the ear skin had significantly thickened in comparison with day 0 (**Figure 5B**). Thus, immunoproteasome inhibition started when disease symptoms were already present, which mimics a therapeutic setup. Daily treatment with ONX 0914 or vehicle was continued until day 7 post first IMQ application (**Figure 5A**). The analysis of IL-17A levels in the serum revealed a significant increase of IL-17A in the IMQ-treated mice compared to vaseline-treated mice, while ONX 0914 treatment significantly reduced the IL-17A levels in the serum to values similar to vaseline-treated control mice (**Figure 5C**). As depicted in **Figure 5A** we could visually observe a reduction of the IMQ-induced lesions after immunoproteasome inhibition. Indeed, both thicknesses of the ear and the back were significantly reduced starting on day 6 (**Figure 5D**). The inflammation scores were reduced in both the ear and the back. However, the reduction of the skin lesions seemed to be more prominent in the ear. Furthermore, the hematoxylin-eosin sections of the ear and back (**Figure 5E**) demonstrated a visual reduction of the tissue thickness and local parakeratosis. An evident reduction of rete ridges, which are considered a main hallmark of psoriasis, can be observed on the back of ONX 0914-treated mice.

**Figure 6:**
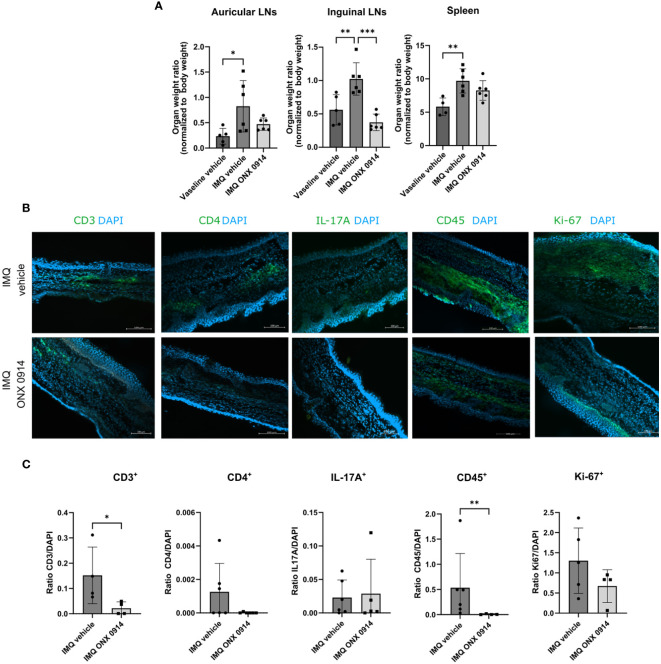
Immunoproteasome inhibition normalizes the weight of dLNs and ameliorates the inflammatory infiltrate in IMQ-induced psoriasis-like inflammation. IL-17A-GFP mice were treated as described in **Figure 5A**. **(A)** The dLNs and spleens were harvested after 8 days of treatment with IMQ/vaseline. On the γ-axis, the organ weight normalized to the body weight is depicted. Data (vaseline vehicle n = 4-5, IMQ vehicle, and ONX 0914 n = 6) was pooled from two independent experiments and analyzed by a one-way ANOVA followed by a Šidák test. **(B)** Representative images of ear cryosections that were stained with anti-CD3, anti-CD4, anti-CD45, anti-Ki67 antibodies or IL-17A (all in green), and DAPI (in blue). The scale bar is 100 μm **(C)** The positive signal was quantified with ImageJ. On the γ-axis, the ratio of the fluorescence signal to DAPI is depicted. Data (n = 4-6) were pooled from 2 independent experiments and statistically analyzed by unpaired t-test or Mann-Whitney test. All values represent mean ± SD. * p < 0.05, ** p <0.01, and *** p < 0.001.

We also observed that dLNs in IMQ-treated mice were heavier (**Figure 6A**). Even though no significant difference was observed for the auricular LNs, we could observe a normalization of the weight in the inguinal LNs of the mice treated with ONX 0914. Although the weight of the spleen was increased after IMQ application, ONX 0914 treatment had no influence.

The recruitment of IL-17A cells to the inflamed areas was analyzed by tracking the expression of GFP on the IVIS Spectrum *in vivo* imaging system. We could detect a significant increase in the GFP signal in the ear and back of IMQ-treated mice (**Supplementary Figure 5**), which suggests that this method can be used to track *in vivo* recruitment of IL-17A^+^ cells to the skin. We detected a lower intensity of the GFP signal on day 8 post first IMQ-treatment in the ear of ONX 0914-treated mice. On the back, no difference between vehicle- and ONX 0914-treated mice could be observed. Additionally, we analyzed the inflammatory infiltrate in the ear by fluorescence microscopy in the IMQ-induced psoriasis model and quantified it (**Figures 6B, C**). Several immune populations were detected in the ear tissue, of which CD45^+^ and CD3^+^ cells were significantly reduced after treatment with ONX 0914. For CD4^+^ and Ki67^+^ a tendency to lower numbers could be observed. Taken together, similar to flow cytometry experiments (**Figure 3**) lower inflammatory infiltrates could be detected by fluorescence microscopy in ONX 0914- treated mice.

## 4 Discussion

During the last decades, intensive research on psoriasis pathogenesis has been translated into the development of potential therapies (31). However, the inconsistency in patient responses (32) and the high rate of psoriasis that remains untreated (33) highlight the need for new and effective treatments. In this study, we demonstrate the effective use of the immunoproteasome inhibitor ONX 0914 in reducing tissue thickness, inflammatory infiltrate, and skin damage in both Card14-mediated and IMQ-induced psoriasis.

Even though the pathogenesis of psoriasis is not fully understood, it is accepted that reactive-oxygen species (ROS) and oxidative stress contribute to disease progression (34). The resulting protein carbonylation, which was detected in patients with psoriasis (35), is irreversible and requires defective proteins to be degraded in order not to disrupt cellular metabolism (36). Such proteins are degraded mainly by the proteasome (37), which is dysregulated in many diseases (38). The analysis of skin lesions revealed that the expression of the 26S proteasome were increased and mainly detected in inflammatory clusters infiltrating the dermis (15). These results strongly support the rationale of treating psoriasis with proteasome inhibitors.

Immunoproteasome inhibitors have been widely used to treat inflammatory diseases in pre-clinical animal models (39). Therapy with broad spectrum proteasome inhibitors were effective in the treatment of psoriasis in the murine SCID-hu model (40) by reducing T cell activation. Although the proteasome inhibitor bortezomib was efficacious in the thioglycolate-induced MCP-1 production model, it exacerbated symptoms in the IMQ-induced psoriasis model (41). In humans, broad spectrum proteasome inhibitors have rather severe side effects, such as anemia, thrombocytopenia, and neutropenia, limiting its therapeutic applicability for psoriatic diseases. However, due to the expression of immunoproteasomes in hematopoietic cells the immunoproteasome inhibitors have fewer toxic side effects (42). Interestingly, the immunoproteasome inhibitor PKS3053 prevented the induction of several IFN-regulated genes and the pro-inflammatory cytokines TNF and IL-1β to tape stripping (43) in a mouse model for atopic dermatitis (44).

Psoriasis is a complex disease that cannot be fully mimicked in animal models. For this reason, we employed two distinct animal models (one chronic and one acute) for testing the efficacy of the immunoproteasome inhibitor ONX 0914. In both, the Card14- and the IMQ-model, we observed the amelioration of physical manifestations of psoriasis in ONX 0914-treated mice. Interestingly, skin cell replacement takes place every 28-30 days in healthy human individuals. However, the turnover is increased to 4-7 days in psoriatic patients (45). Therefore, we analyzed cell proliferation by detecting Ki-67^+^ cells in the dermis and epidermis of *Card14ΔE138* ^+/-^ mice. Even though we did not detect a significant alteration of Ki-67^+^ cells after treatment with ONX 0914 (**Figure 3**), we found cell counts of CD45^+^, CD3^+^, CD4^+^, CD4^+^IL-17A^+^ and CD11b^+^ Ly6G+ to be markedly reduced after treatment.

Cytokine members of the IL-23/IL-17 family are critical in the development of autoimmunity and psoriasis (46). IL-23 activates Th17 cells through the STAT3 pathway and promotes the production of IL-17A, IL-22 and TNF, which induce the proliferation of keratinocytes expressing the IL-22 receptor (47). We observed that CD4^+^ IL-17A^+^ cells were significantly increased in the spleen of *Card14ΔE138* ^+/-^ mice and subsequently diminished after immunoproteasome inhibitor treatment (**Figure 1**). However, such upregulation of the IL-17A cells in the spleen could not be detected in IMQ-treated mice (data not shown). Cutaneous inflammation is not a problem solely related to skin, but the release of several inflammatory products into systemic circulation can affect other organs resulting in comorbidities (48). Interestingly, IL-17A is responsible for the formation of amyloidosis in both the liver and the spleen (49), a disorder in which abnormal proteins accumulate. Additionally, IL-17-related cytokines play an important function in the formation of microabscesses by neutrophils through “connection to IκB kinase and stress-activated protein kinases” signaling into the keratinocytes (50). In line with this, and contributing to the reduced disease symptoms in our study, we observed a reduction of neutrophils (CD11b^+^Ly6G^+^) accompanied by a normalization in the cell counts of several other immune cell populations in the skin of *Card14ΔE138* ^+/-^ mice treated with ONX 0914 (**Figure 3**).

γδ^+^ T cells are a particular population of T lymphocytes. Even though most of the studies have focused on αβ^+^ T cells, there is increasing evidence that aberrantly activated γδ^+^ T cells play an important role in the pathogenesis of autoimmune disorders, such as psoriasis (51). IL-23 predominantly stimulates dermal γδ^+^ T cells to produce IL-17 that leads to disease progression (29). Since both αβ^+^ and γδ^+^ T cell population have the ability to secrete IL-17A and IL-22 (52) we investigated these populations in the skin samples of diseased *Card14ΔE138* ^+/-^ mice (**Figure 4**). We observed that αβ^+^ T cells are the main producers of IL-17A in the skin *of Card14ΔE138* ^+/-^ mice, which is in line with prior analysis (53). Little IL-22 was secreted by γδ^+^ T cells in *Card14ΔE138* ^+/-^ mice. Interestingly, ONX 0914 treatment reduced the percentage of αβ^+^ cells and αβ^+^ cells secreting IL-17A, whereas it increased the frequency of γδ^+^ T cells and IL-22 production. IL-22 is primarily involved in preservation of the mucosal barrier and protection of the host from microbial parasites in the skin (54). The anti-apoptotic effects of IL-22 (55) together with the capability to promote regeneration and proliferation highlights IL-22´s ability to promote healing and skin repair (56). Whether γδ^+^ T cells may have a protective function by increased production of IL-22 is currently unknown. Remarkably, we observed a double positive γδ^+^ αβ^+^ T cell population in the ear tissue (**Figure 4B**). Several non-common αβ/γδ TCRs have been previously reported (57–60) and suggested to be produced as unusual gene rearrangements. Recently, Reitermaier et al. discovered that αβγδ double positive T cells are present in fetal human samples and are essential in the skin development and immunity (61). Whether these αβ^+^γδ^+^ cells play a relevant role in our disease model is currently unknown.

Taken together, this study shows that ONX 0914 significantly reduced the skin thickness and pathological features in two different murine model of psoriasis. The analysis of skin samples revealed normalization of pro-inflammatory cytokines and cell populations that contribute to the pathogenesis of psoriasis. Moreover, the reduction of αβ^+^ T cells was accompanied by a significant shift in the IL-17A and IL-22 secretion. Altogether, this study highlights the potential therapeutic use of immunoproteasome inhibitors in the treatment of psoriasis.

## Data availability statement

The original contributions presented in the study are included in the article/**Supplementary Material**. Further inquiries can be directed to the corresponding author.

## Ethics statement

The animal study was reviewed and approved by Regierungspräsidium Freiburg.

## Author contributions

Conceptualization: MB and MDRO, Investigation and formal analysis: MDRO, Funding acquisition: MB, Providing mice: MM, Supervision: MB, Writing: MDRO, Review and editing: MM and MB. All authors contributed to the article and approved the submitted version.
