# Corrigendum: Immunoproteasome inhibition attenuates experimental psoriasis

**DOI:** 10.3389/fimmu.2023.1335691

**Published:** 2024-01-16

**Authors:** Marta del Rio Oliva, Mark Mellett, Michael Basler

**Affiliations:** ^1^ Division of Immunology, Department of Biology, University of Konstanz, Konstanz, Germany; ^2^ Department of Dermatology, University Hospital Zürich (USZ), Zürich, Switzerland; ^3^ Faculty of Medicine, University of Zürich (UZH), Zürich, Switzerland; ^4^ Biotechnology Institute Thurgau at the University of Konstanz, Kreuzlingen, Switzerland

**Keywords:** immunoproteasome inhibition, psoriasis, CARD14, imiquimod, ONX 0914

In the published article, there was an error in **Figure 6** as published. In **Figure 6B** the representative microscopy images for CD3 IMQ vehicle and IMQ ONX 0914 were mistakenly switched and the representative image for IL-17A IMQ vehicle was incorrectly incorporated. The corrected **Figure 6** and its caption appear below.

**Figure 6:**
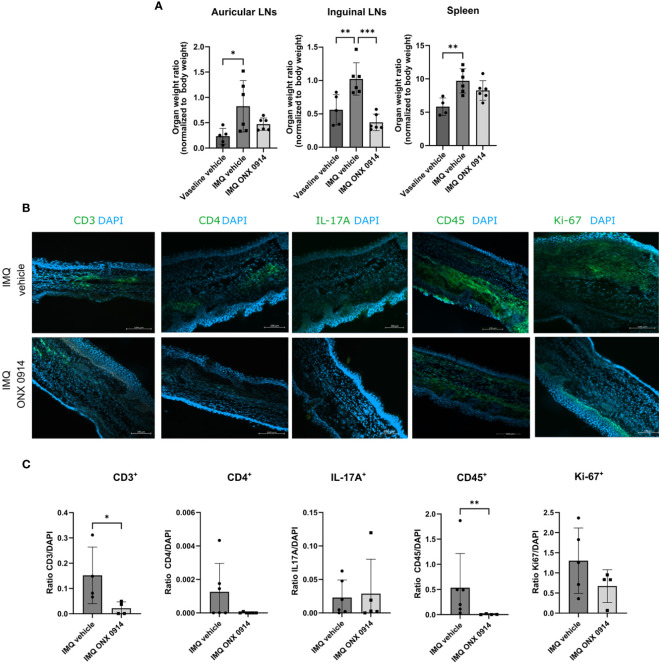
Immunoproteasome inhibition normalizes the weight of dLNs and ameliorates the inflammatory infiltrate in IMQ-induced psoriasis-like inflammation. IL-17A-GFP mice were treated as described in Figure 5A. **(A)** The dLNs and spleens were harvested after 8 days of treatment with IMQ/vaseline. On the γ-axis, the organ weight normalized to the body weight is depicted. Data (vaseline vehicle n = 4-5, IMQ vehicle, and ONX 0914 n = 6) was pooled from two independent experiments and analyzed by a one-way ANOVA followed by a Šidák test. **(B)** Representative images of ear cryosections that were stained with anti-CD3, anti-CD4, anti-CD45, anti-Ki67 antibodies or IL-17A (all in green), and DAPI (in blue). The scale bar is 100 μm **(C)** The positive signal was quantified with ImageJ. On the γ-axis, the ratio of the fluorescence signal to DAPI is depicted. Data (n = 4-6) were pooled from 2 independent experiments and statistically analyzed by unpaired t-test or Mann-Whitney test. All values represent mean ± SD. *p < 0.05, **p <0.01, and ***p < 0.001.

The authors apologize for this error and state that this does not change the scientific conclusions of the article in any way. The original article has been updated.

