# Supplementary material for: Immunoproteasome inhibition attenuates experimental psoriasis
Source: Front Immunol. 2022 Dec 14;13:1075615. doi: 10.3389/fimmu.2022.1075615 (PMC9798438; doi:10.3389/fimmu.2022.1075615)
Supplement: Supplementary file 1 [file DataSheet_1.docx]

Supplementary Material

| Gene | Sequence |
| --- | --- |
| *Ccl20* fwd | 5´GAGCTATTGTGGGTTTCACAAGAC3´ |
| *Ccl20* rev | 5´ACTCTTAGGCTGAGGAGGTTCAC3´ |
| *Il17a* fwd | 5´ CCGCAATGAAGACCCTGATAGAT3´ |
| *Il17a* rev | 5´AGAATTCATGTGGTGGTCCAGC3´ |
| *Il17c* fwd | 5´CTGGAAGCTGACACTCACG3 |
| *Il17c* rev | 5´GGTAGCGGTTCTCATCTGTG3´ |
| *Tnf* fwd | GCCTCCCTCTCATCAGTTCT3´ |
| *Tnf* rev | 5´CACTTGGTGGTTTGCTACGA3´ |
| *Hprt* fwd | 5´GTCTTCCTACCCCCAATG3´ |
| *Hprt* rev | 5´TGTCATCATACTTFFCAG3´ |
| *Il6* fwd | 5´ACACATGTTCTCTGGGAAATCGT3´ |
| *Il6* rev | 5´AAGTGCATCATCGTTGTTCATACA3´ |
| *Cxcl2* fwd | 5´AAGTTTGCCTTGACCCTGAA3´ |
| *Cxcl2* rev | 5´AGGCACATCAGGTACGATCC3´ |
| *Il22* fwd | 5´TTTCCTGACCAAACTCAGCA3´ |
| *Il22* rev | 5´TCTGGATGTTCTGGTCGTCA3´ |
| *Il23* fwd | 5´AATAATGTGCCCCGTATCCA3´ |
| *Il23* rev | 5´CTGGAGGAGTTGGCTGAGTC3´ |

**Table 1:** Primers used for real-time RT-PCR.

| **Antigen** | **Fluorochrome** | **Clone** | **Supplier** |
| --- | --- | --- | --- |
| CD4 | BV605 | GK1.5 | Biolegend |
| CD4 | FITC | GK1.5 | Biolegend |
| CD45 | FITC | 30-F11 | Biolegend |
| CD45 | BV421 | 30-F11 | Biolegend |
| CD45 | PE | 30-F11 | eBioscience |
| CD8 | PE-Cy7 | 53-6.7 | Biolegend |
| CD19 | APC | 6D5 | Biolegend |
| CD3 | FITC | 145-2C11 | Biolegend |
| CD11b | PE-Cy7 | M1/70 | Biolegend |
| Ly6G | AlexaFLuor700 | 1A8 | BD Biosciences |
| IL-17A | APC | ebio17B7 | eBioscience |
| IL-17A | PE | Ebio17B7 | invitrogen |
| IL-22 | PerCP/Cy5.5 | Poly5164 | Biolegend |
| CD11c | PE | HL3 | BD Pharmingen |
| βTCR | PE-Cy7 | H57-597 | Biolegend |
| TCR γ/δ | BV605 | GL3 | Biolegend |

**Table 2**: Antibodies used for flow cytometry and immunofluorescence.

**
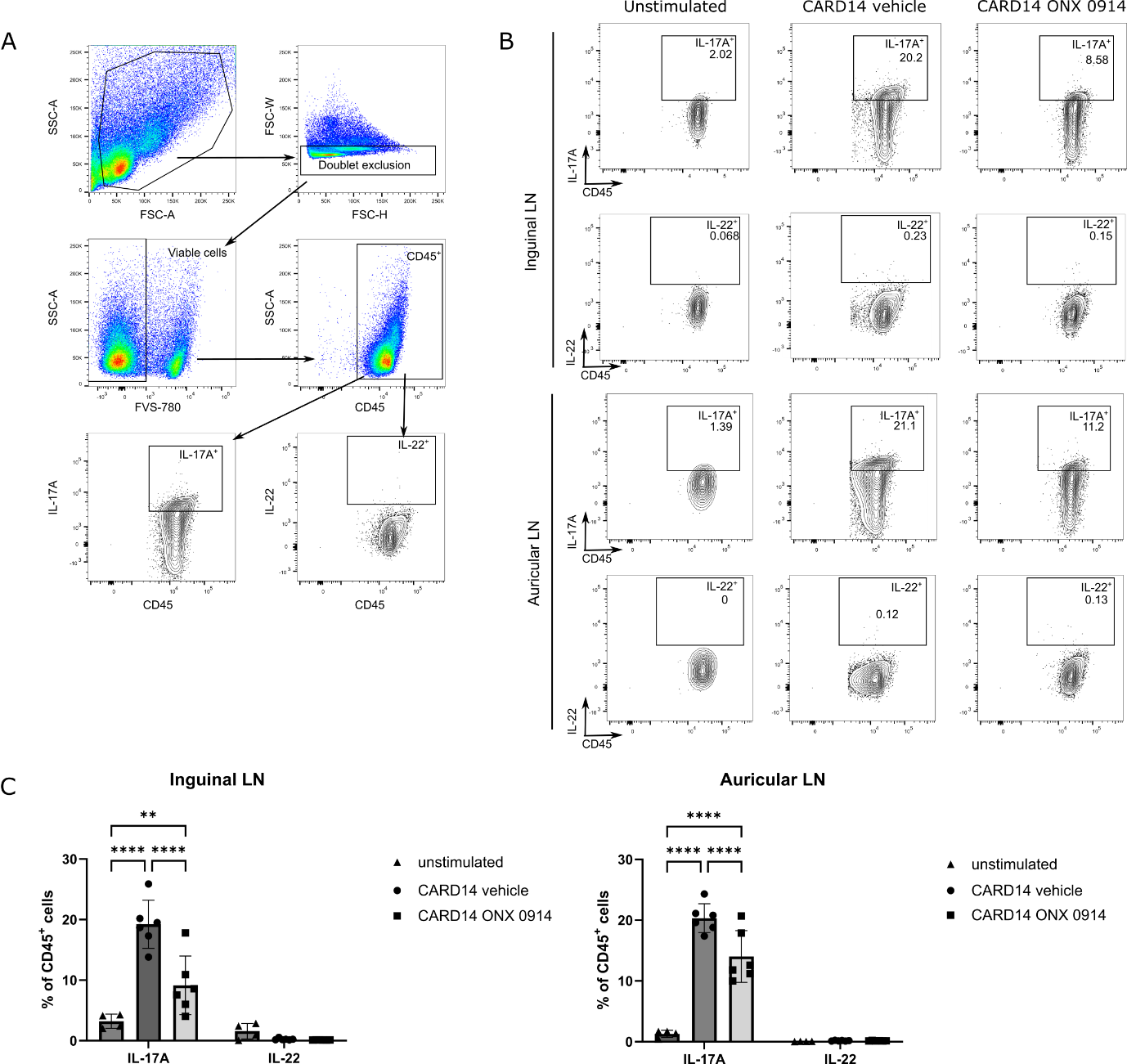
Supplementary Figure 1: IL-17A and IL-22 secretion in dLNs.** *Card14ΔE138*^+/-^ mice were treated as stated in Fig. 1A. On day 20, the inguinal and auricular LNs were collected and stimulated with PMA/ionomycin and an ICS for IL-17A and IL-22 was performed. (A) Gating strategy of IL-17A^+^ and IL-22^+^ cells was performed after doublet and dead cell exclusion and pre-gating on CD45^+^ cells (B) Representative dot plots. (C) On the γ-axis, the percentage of IL-17A^+^ and IL-22^+^ cells from the CD45^+^ pool is depicted. Data from unstimulated CARD14 vehicle mice (n=4), CARD14 vehicle (n=6) and CARD14 ONX 0914 (n=6) were pooled from 2 independent experiments and statistically analyzed by 2-way ANOVA followed by a Bonferroni post hoc test. All values represent mean ± SD.

**
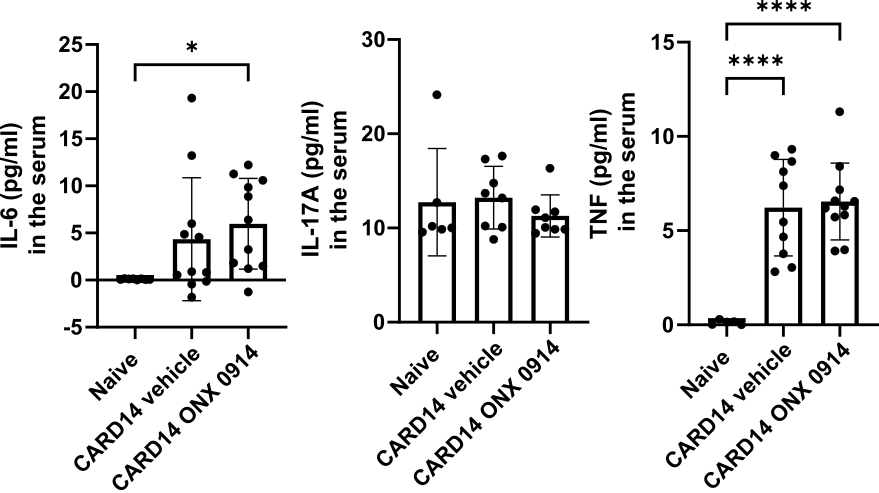
**

**Supplementary Figure 2.** **Cytokine levels in the serum of *Card14ΔE138^+/-^* mice*.*** *Card14ΔE138*^+/-^ mice were treated as stated in Figure 1A. The concentrations of IL-6, IL-17A and TNF in the serum were analyzed by ELISA. On the γ-axis, serum cytokine in pg/ml is depicted. Data (n=8-11) were pooled from 2 independent experiments and statistically analyzed by one-way Anova followed by a Tukey test. All values represent mean ± SD.


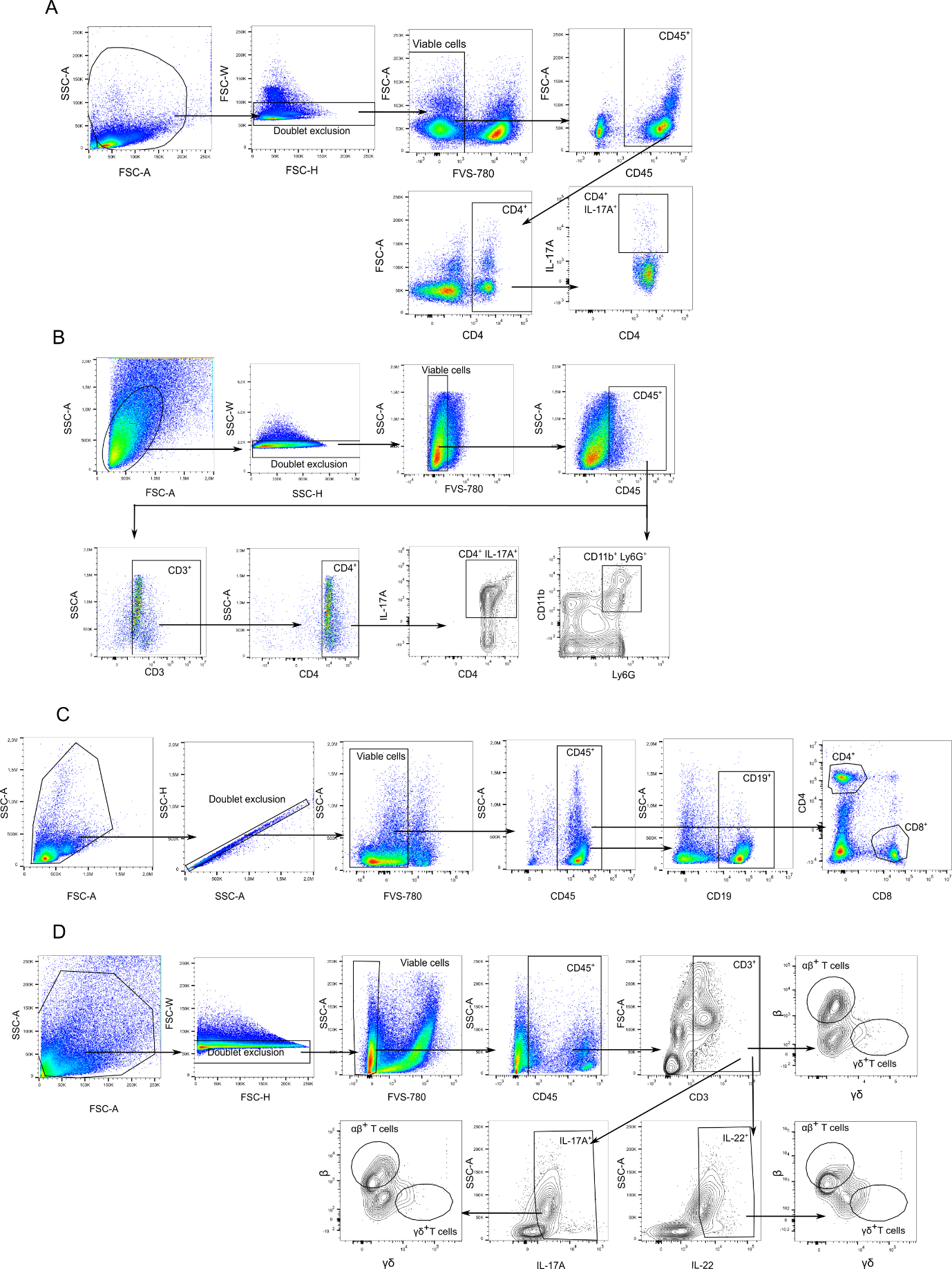


**Supplementary Figure 3.** **Gating strategy for flow cytometry analysis of the ear and spleen of *Card14ΔE138*^+/-^ mice**. The mice were treated as stated in Figure 1A. (A) The spleen of ONX 0914 and vehicle-treated mice was collected on day 20 and stimulated with PMA/ionomycin for 4 hours, followed by ICS. The IL-17A^+^ CD4^+^ cells were analyzed after doublet and dead cell exclusion. The splenocytes were pre-gated on CD45^+^ cells. (B) A single cell suspension of the ear was prepared after 20 days of ONX 0914 or vehicle treatment and the CD45^+^, CD3^+^, CD4^+^, CD11b^+^Ly6G^+,^ and CD4^+^IL-17A^+^ populations were analyzed by flow cytometry. The gating of CD45^+^, CD11b^+^Ly6G^+^, CD3^+^, CD4^+^, and CD4^+^IL-17A^+^ cells was performed after doublet and dead cell exclusion and pre-gating on CD45^+^ cells. CD4^+^IL-17A^+^ cells were pre-gated on CD3^+^CD4^+^ cells. (C) The spleen was analyzed for CD45^+^, CD8^+^, CD4^+^, and CD19^+^ after doublet and dead cell exclusion and pre-gating on CD45^+^ cells. (D) The αβ^+^ and γδ^+^ cells were analyzed in the ear tissue of Card14ΔE138^+/-^ mice by stimulating the cells with PMA/ionomycin for 4 hours followed by ICS for IL-17A and IL-22. The cells were pre-gated on CD45^+^ CD3^+^ cells or pre-gated on CD45^+^ CD3^+^ cells and IL-17A or IL-22.


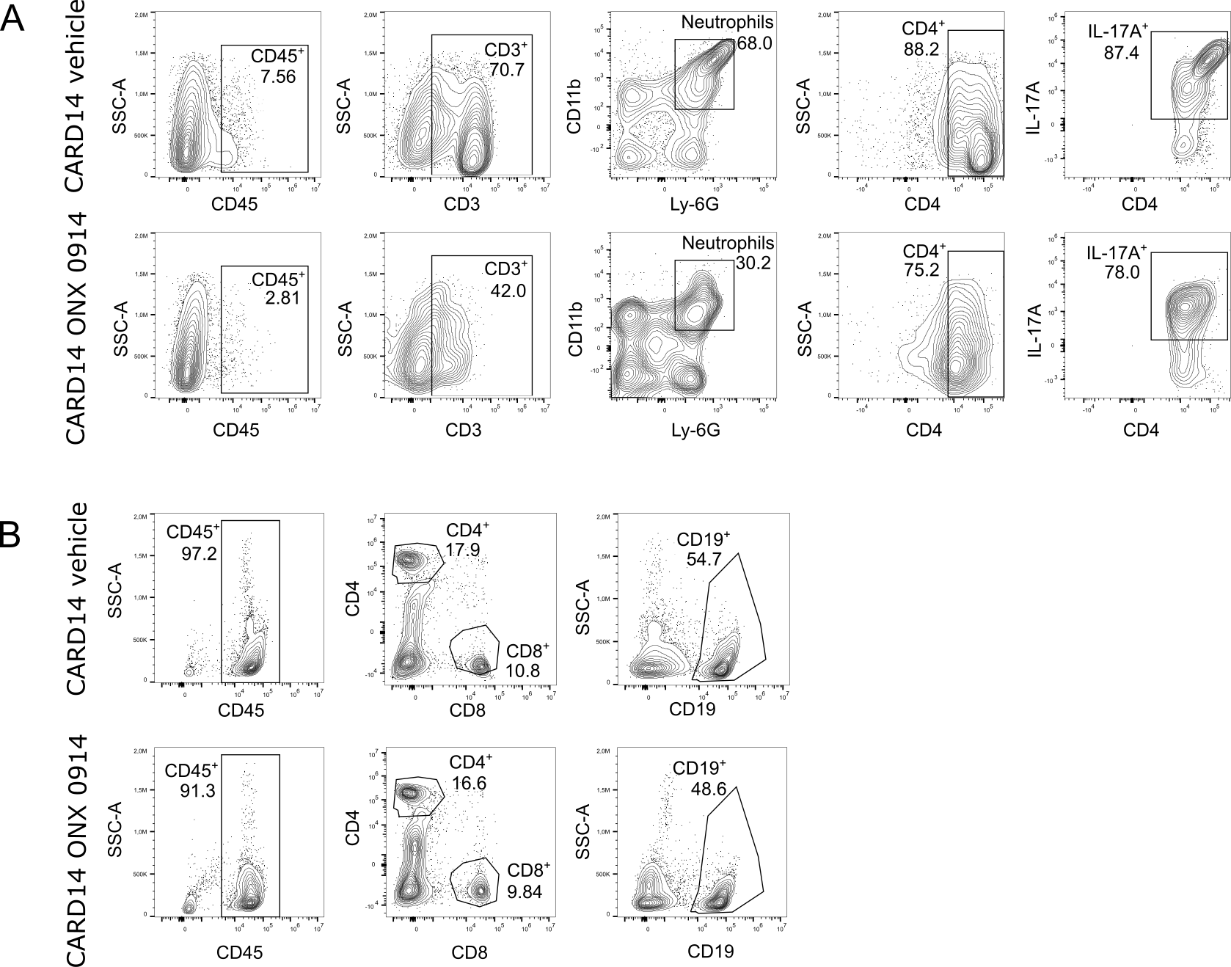


**Supplementary Figure 4. Representative flow cytometry dot plots of the ear and spleen of *Card14ΔE138*^+/-^ mice**. The mice were treated as stated in Figure 1A. (A) A single cell suspension of the ear was prepared and the CD45^+^, CD11b^+^Ly6G^+^, CD3^+^, CD4^+^, and CD4^+^IL-17A^+^ populations were analyzed. The gating strategy is depicted in Supplementary Figure 3B. (B) The spleen was analyzed for CD45^+^, CD8^+^, CD4^+^, and CD19^+^. The gating strategy is depicted in Supplementary Figure 3C.


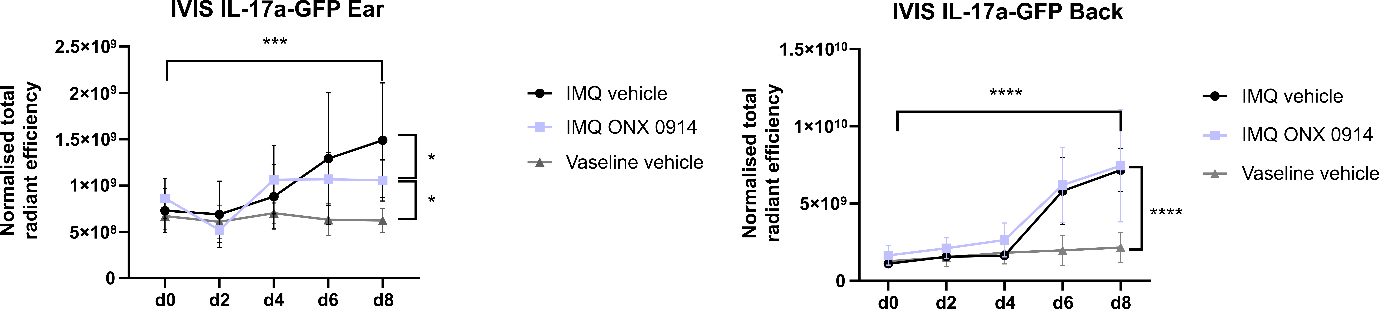


**Supplementary Figure 5**. **IVIS acquisition in the IMQ-induced model.** IL-17A-GFP mice were treated as stated in Fig. 4A. On alternate days, the recruitment of IL-17A^+^ cells to the skin was analyzed with an IVIS imaging system. The radiant efficiency of the ear and the back was calculated and normalised to the background. On the γ-axis, the normalized radiant efficiency is depicted. Data (n=6) were pooled from 2 independent experiments and statistically analyzed by 2-way Anova followed by a Fisher´s LSD test. All values represent mean ± SD.
